# A novel 3D ray launching technique for radio propagation prediction in indoor environments

**DOI:** 10.1371/journal.pone.0201905

**Published:** 2018-08-07

**Authors:** Tan Kim Geok, Ferdous Hossain, Alan Tan Wee Chiat

**Affiliations:** Faculty of Engineering Technology, Multimedia University, Ayer Keroh Lama, Melaka, Malaysia; Nanjing University of Information Science and Technology, CHINA

## Abstract

Radio propagation prediction simulation methods based on deterministic technique such as ray launching is extensively used to accomplish radio channel characterization. However, the superiority of the simulation depends on the number of rays launched and received. This paper presented the indoor three-dimensional (3D) Minimum Ray Launching Maximum Accuracy (MRLMA) technique, which is applicable for an efficient indoor radio wave propagation prediction. Utilizing the novel MRLMA technique in the simulation environment for ray lunching and tracing can drastically reduce the number of rays that need to be traced, and improve the efficiency of ray tracing. Implementation and justification of MRLMA presented in the paper. An indoor office 3D layouts are selected and simulations have been performed using the MRLMA and other reference techniques. Results showed that the indoor 3D MRLMA model is appropriate for wireless communications network systems design and optimization process with respect to efficiency, coverage, number of rays launching, number of rays received by the mobile station, and simulation time.

## Introduction

The rapid growth of wireless communications network systems, all over the world within last few decades has become an emergent need of suitable and efficient computerized tools for radio wave propagation prediction in various environments [1]. The wireless communication system for the indoor scenario, which is connected with wireless local area networks and personal communication are increasing rapidly. Therefore, Radio wave propagation prediction model plays a vital role in the analysis of wireless communication system. The application of smart radio propagation modelling to assist smart city design, 4G mobile networks further upgradation, Internet of Things, and the evaluation of higher frequency based 5G network system [2]. Radio propagation is a smart way of analysing and predicting the signal details [3]. Even though direct measurements enable accurate evaluation of onsite performance, but it requires a considerable amount of time and efforts. Contrariwise, computerized simulation’s solution tool is very much dynamic and low-cost and producing perfect results [4]. Therefore, a computerized simulation tool that could characterize the wireless channel from the indoor-outdoor plans and material properties would be a good solution. Significant knowledge about the propagation channel modeling is the prerequisite to analysis, design, and deployment of the wireless communication system. From the literature, review observed that radio propagation channels categorization for various scenarios has become an important research topic [5–7]. In this circumstance, it is mandatory to minimize computational time and requisite resource demands for radio wave propagation prediction and optimum base-station position configuration. Compatible and faster radio propagation channel modeling mainly depends on frequency, and types of signals with respect to the surrounding environment [8].

The indoor radio propagation is not hampered by weather controlling parameters, such as heavy rains, floods, clouds, or snowfall as outdoor; nevertheless, the indoor building-walls, items of furniture, windows, doors, and other household items can affect it. These indoor elements need to take into account for accurate propagation prediction; moreover, the environment containing the mentioned obstacles those transmitter (Tx) signals will be reached to the receiver (Rx) via multipath channels because of reflections [9].

Geometric Optics (GO) and Uniform Theory of Diffraction (UTD) based ray tracing (RT) is widely used to model the channel of indoor radio wave propagation [10]. Currently, most of radio propagation researchers are highly recommending RT for propagation modeling because it is more suitable for radio wave propagation modeling [11]. The RT mainly consists three steps such as ray launching (RL), ray path searching and ray receiving [12]. The RL is the process of the shooting of straight rays in the possible all directions around the entire space. The rays launch from the base stations like Tx to the mobile station like the Rx with respect to the theory of GO, and UTD. Then trace the total path of each ray considering several additional propagation scenarios, like transmission, reflection, and diffraction [13]. Finally, in the ray receiving step, estimate whether the ray has an intersection with Rx point or not by using the Rx ball area sphere.

The first point of RT is denoted as RL, where RL technique offer a noble tradeoff between accuracy and computational cost [14]. However, for massive complicated environment in which lots of potential mobile stations and base stations can be positioned, the conventional RL demonstrates huge computational time and coverage limitations [15, 16]. In conventional 3D RL methods, no any probable zone is applied for potential Rx positions in a given scenario. It normally uses forward-RT with the specific range of vertical angle (θ) and azimuth angle (Φ) resolution, which mention as shooting and bouncing rays (SBR) [17–21]. Therefore, for the 3D radio wave propagation prediction, the large number of rays are launched from the Tx point to all directions in of space. In this circumstance, the rays intersect with considerable obstacles (e.g. wall and wood) respectively by reflection and continues the ray propagation throughout the whole scenario [22]. At the end, a ray may reach at the targeted Rx point with respect to intersect with the Rx reception sphere, or it may be lost finally from the whole scenario because of does not intersect with the Rx reception sphere. The main weakness of SBR method is that the zone of the Rx sphere is undefined and thus it needs to lunch rays in all angles from Tx. It's strongly hampering for computational time, accuracy and coverage.

In order to reduce computational time, without sacrificing accuracy, ray launching need to be optimized by using this MRLMA RL algorithm in simulation improved all the limitations significantly.

The paper is organized as follows. Firstly, in section two the simulation environment-configuration is explained. Secondly, 3D MRLMA algorithm discussed in section three. Thirdly, compared the result with existing method’s result in section four. Finally, draw the conclusions in section five.

### Simulation environment configuration

In this research, the proposed MRLMA algorithm has been implemented in in-house developed simulator. The simulator was developed using programing language visual C# (WPF, Visual Studio 2017, version: 15.5.2) an object-oriented programming language and database is SQL server 2017 standard edition. The common parameters that was actively used in proposed and convention algorithm is mention below in Table 1 [23, 24].

**Table 1:** Simulation parameter.

Item	Value
Frequency	6 GHz
Transmitter power	30 dBm
Dimension(in Pixel)	3000 * 3000
Number of pixels per meter	40 Pixels
Maximum No. of Reflections	128
Base Station Height	1.5 meter
Ray Thickness	1 Pixel

The simulator can perform an intersection for a ray and obstacles. Although the MRLMA determine the intersection angles are different for different obstacles, the same code block may be called to perform the intersection until simulation finished. Simulations have been performed by using 64-bit windows server (Y0M88AA#UUF) which configuration are mentioned. OS Name MS Windows 10. Processor AMD Ryzen 7 1800X Eight-Core CPU, 3600 MHz, 8 Core, 16 Logical CPU(s). Installed random-access memory (RAM) 16.0 GB. Graphics card AMD-Radeon™-RX 580 (4 GB GDDR5).

### 3D MRLMA algorithm

The development of 3D MRLMA RL algorithm has four steps:

- Step I: Scenario creation
- Step II: 3D ray-launching only for calculation to identify the Rx zone.
- Step III: More ray launching vertical angles define in identified zone.
- Step IV: Predefine vertical angle wise 3D ray launching.

In step I, the different 3D scenario on the layout is created by considering several obstacles, mobile stations, base stations and the whole components of the environment. Configurable parameters such as operational frequency, transmitter power, the maximum number of reflections, and the dimension can be modified in the method base on the environment. In order to gain satisfactory output values, it is very significant to take into consideration not only the configurable parameters values which output is more accurate but also the parameters values which has the direct impact on computational time, because which can be very important for a complex scenario.

In step II, RL and it propagation along the scenario intersecting with the obstacles, because of physical sensations such as reflection, refraction, and diffraction. The algorithm runs the code block in a recursive manner.

**Fig 1:**
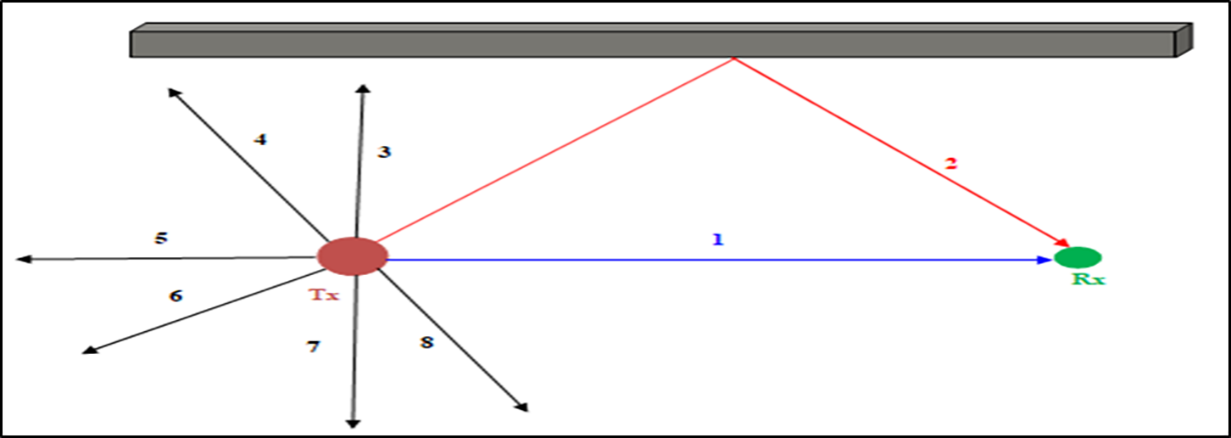
Indoor ray launching.

The Tx launches rays in all directions, and the Rx receive the LOS ray 1 and the NLOS ray 2, while the other rays are not received. Considering the scenario from the Fig 1 number of ray launching can reduce and that has no effect on the accuracy of the result because the rays launched from the zone of ray 3, 4, 5, 6, 7, and 8 does not receive to Rx. So in this area, launching rays in higher resolution vertical angle difference is not related to the purpose, also it has the very small effect on the results. From this ray launching base on the scenario in vertical angle θ = π/60 difference and calculated the successive angles θ those are contributed to Rx.

In step III, here every successive vertical angle wise RL zone makes wider by adding an immediate point of more angles in both forward and backward of identified vertical angles.

**Fig 2:**
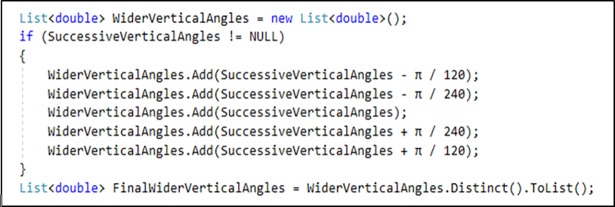
Vertical angles wider C# code.

For every successive angle which was identified in step 2 are recursively used in the Fig 2 mention code block to pinpoint more ray in the potential zone. Here those additional vertical angles are very close to successive vertical angle points.

In step IV, RL start with the predefined vertical angle that received from step III. This RL ensures higher level of accuracy with respect to predefine potential zone from these update vertical angles and get more accurate results. Maximum number RL rays may contribute at the mobile station ball of Rx.

## Results and discussion

In the result discussion, an indoor office layout has been selected for simulation. Three unique scenarios have been created with respect to place the Tx and different number of Rx in different places. For the specific layout scenario, ray has been launched using SBR and MRLMA methods. All the relevant configurable parameters were same for both simulations methods. In the result, simulation ID, Rx unique ID, the number of rays received by Rx, total simulation time, and the total number of launching rays are presented consecutively. For SBR vertical and azimuth angles regulation differences is 1 degree which is use for ray launching [25].

The result and discussion section is divided into three parts. The first part discusses, like Tx placed in the center from the point of view of the scenario 1. The second part discusses, Tx placed in the right side from the point of view of the scenario 2. The third part discusses, Tx placed in the left side from the point of view of the scenario 3. The term evaluation is used interchangeably with numbers of rays received in Rx, the total number of ray launch, and simulation time, which are the active dimension of overall performance comparison.

**Fig 3:**
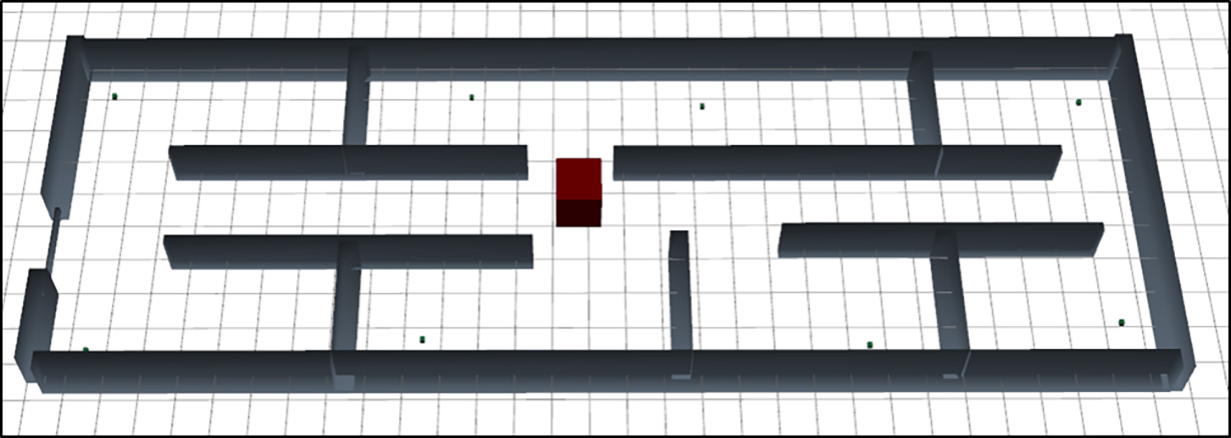
3D graphical representation of scenario 1.

This layout is designed base on typical office’s first floor considering seven rooms. One base station is placed in the center of the floor. Six Rx placed in the six rooms and two placed in the larger room. Fig 3 express the graphical design of the mentioned layout scenario 1.

**Fig 4:**
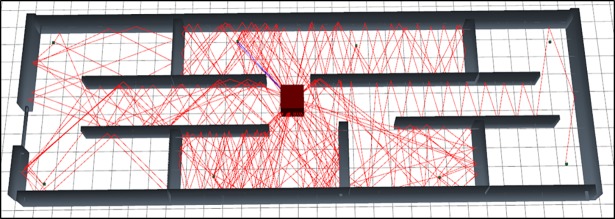
3D graphical view of scenario 1 simulation with SBR.

The Fig 4 represents the graphical output of the simulation of scenario 1 using SBR method. As per visual representation in this Fig 4 express that, it bearing large numbers of reflections so it’s path loss is very high.

Simulation output data for scenario 1 using SBR is presented in Table 2. As per the Table 2 data, the maximum and the minimum number of rays those reached in the Rx are nine and one. In total forty-two rays contributed to eight Rx where the total number of rays launching is 64800. Total simulation time is 6398.44 (ms).

**Table 2:** Simulation data for scenario 1 using SBR algorithm.

Simulation ID	Mobile Station ID	Ray Received	Simulation Time(MS)	Number of Ray Launched
MMU-FET-20180103160003	17ba2de7-1519-43dc-b346-52d46d16b043	9	6398.44	64800
	2ed67a0f-87d3-407d-99c3-14e280b38e1c	5		
	7f820540-d20d-4fbb-a33f-0591633466d0	3		
	8e05c55b-8ab2-4da8-bb61-3d9088a89f49	4		
	9fd880f3-4110-464f-b952-4c5e45bba8e9	2		
	c260fa34-4024-4d96-bcdf-f44f5a739a13	8		
	ce3ccbd9-ae06-46b1-bcee-44f2e7f6072a	1		
	f8cb1f8f-3065-4586-a8a5-cededb6b1ccd	10		
**Total**		**42**	**6398.44**	**64800**

**Fig 5:**
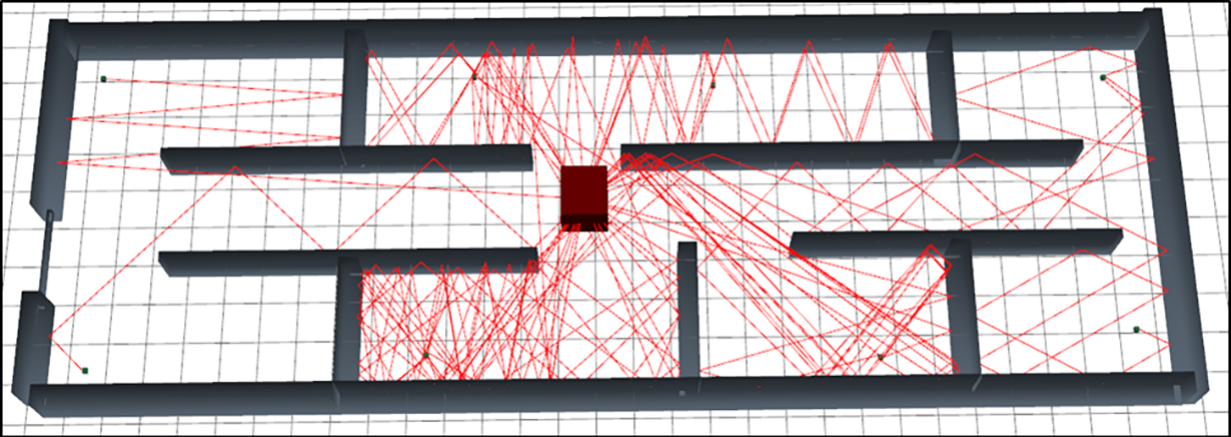
3D graphical view of scenario 1 simulation with MRLMA.

The Fig 5 represents the graphical output of the simulation of scenario 1 using MRLMA method. As per visual representation in this Fig 5 express that, it bearing fewer numbers of reflections so it's path loss is low.

Simulation output data for scenario 1 using MRLMA is presented in Table 3. As per Table 3 data, the maximum and the minimum number of rays that reached in the Rx are seventeen and one. In total forty-nine rays contributed to eight Rx where the total number of rays launching is 26280. Total simulation time is 3549.24(ms).

**Table 3:** Simulation data for scenario 1 using MRLMA algorithm.

Simulation ID	Mobile Station ID	Ray Received	Simulation Time(MS)	Number of Ray Launched
MMU-FET-20180103160056	17ba2de7-1519-43dc-b346-52d46d16b043	11	3549.24	26280
	2ed67a0f-87d3-407d-99c3-14e280b38e1c	5		
	7f820540-d20d-4fbb-a33f-0591633466d0	1		
	8e05c55b-8ab2-4da8-bb61-3d9088a89f49	2		
	b21de9db-acfa-4fc3-9800-0045a5962f8f	3		
	c260fa34-4024-4d96-bcdf-f44f5a739a13	9		
	ce3ccbd9-ae06-46b1-bcee-44f2e7f6072a	1		
	f8cb1f8f-3065-4586-a8a5-cededb6b1ccd	17		
**Total**		**49**	**3549.24**	**26280**

**Fig 6:**
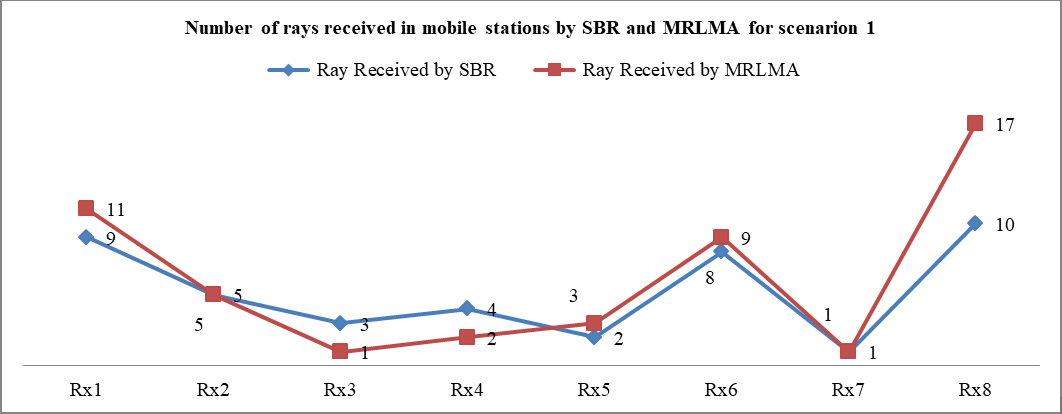
Comparison graph of the number of received rays to Rx by RL using SBR and MRLMA methods for scenario 1.

The Fig 6 shows that the coverage of using MRLMA is better than SBR method because the number of received rays by Rx is higher in the proposed method. From the scenario 1 both simulations data, it is shown that only 40.55% rays launching need to get better coverage in the proposed method compared to the conventional SBR. It also saves 44.52% simulation times.

**Fig 7:**
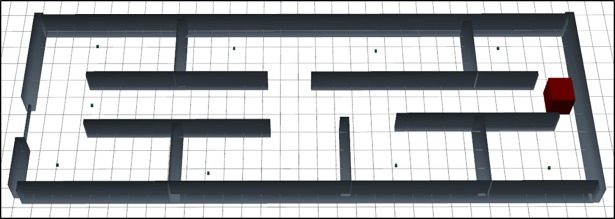
3D graphical representation of scenario 2.

The Fig 7 express the graphical design of the scenario 2 on same layout like Fig 3. One base station is placed on the right side of the floor. Six Rx placed in the six rooms, one is in connection path of room, and two is placed in the larger room.

**Fig 8:**
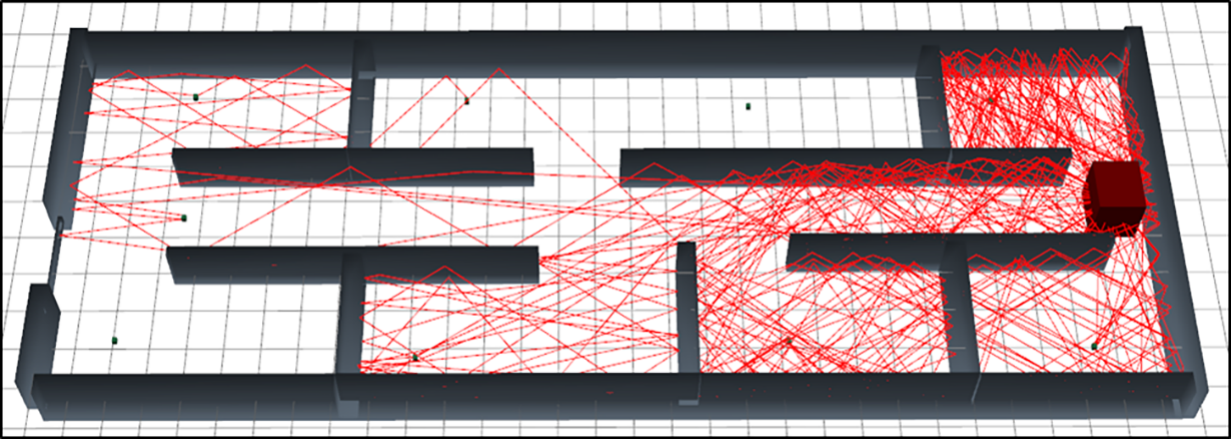
3D graphical view of scenario 2 simulation with SBR.

The Fig 8 represents the graphical output of the simulation of scenario 2 using SBR method. As per visual representation in this Fig 8 express that, it bearing large numbers of reflections so it’s path loss is very high.

Simulation output data for scenario 2 using SBR is presented in Table 4. As per the Table 4 data, the maximum and the minimum number of rays, that reached in the Rx are twenty, and one. In total, fifty three rays contributed among the seven Rx where the total number of rays launching is 64800. Total simulation time is 7530.84 (ms).

**Table 4:** Simulation data for scenario 2 using SBR algorithm.

Simulation ID	Mobile Station ID	Ray Received	Simulation Time(MS)	Number of Ray Launched
MMU-FET-20180103173206	57725d67-e700-4bf7-8757-97c68b19e558	9	7530.84	64800
	58c97142-9b74-47e5-9f75-b78b365141a7	8		
	5a299dc0-c485-45a3-8922-785a2ea2f562	2		
	726ffb8f-80a1-414f-82f7-a86da6e4151d	20		
	79cced51-c8f4-40a4-b5bd-512d99ed95b1	1		
	9c571bd6-8ca7-45be-9cfc-6bd4f708aacb	3		
	a5d2e52b-b087-4f20-b5a4-a6ae2b3b622a	10		
**Total**		**53**	**7530.84**	**64800**

**Fig 9:**
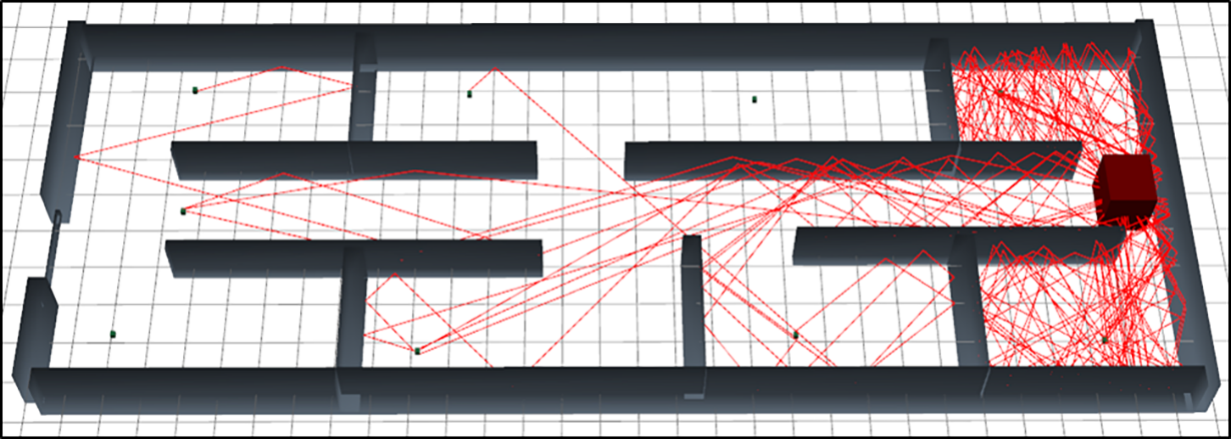
3D graphical view of scenario 2 simulation with MRLMA.

The Fig 9 represents the graphical output of the simulation of scenario 2 using MRLMA method. As per visual representation in this Fig 9 expresses that, it bearing fewer numbers of reflections so it’s path loss is very low.

Simulation output data for the scenario 2 using MRLMA method is presented in Table 5. As per the Table 5 data, the maximum and the minimum number of rays, that reached in the Rx are twenty-six, and one. In total sixty-five rays contributed among the seven Rx where the total number of rays launching is 28080. Total simulation time is 4522.34 (ms).

**Table 5:** Simulation data for scenario 2 using MRLMA algorithm.

Simulation ID	Mobile Station ID	Ray Received	Simulation Time(MS)	Number of Ray Launched
MMU-FET-20180103173305	57725d67-e700-4bf7-8757-97c68b19e558	21	4522.34	28080
	58c97142-9b74-47e5-9f75-b78b365141a7	9		
	5a299dc0-c485-45a3-8922-785a2ea2f562	1		
	726ffb8f-80a1-414f-82f7-a86da6e4151d	26		
	79cced51-c8f4-40a4-b5bd-512d99ed95b1	1		
	9c571bd6-8ca7-45be-9cfc-6bd4f708aacb	3		
	a5d2e52b-b087-4f20-b5a4-a6ae2b3b622a	4		
**Total**		**65**	**4522.34**	**28080**

**Fig 10:**
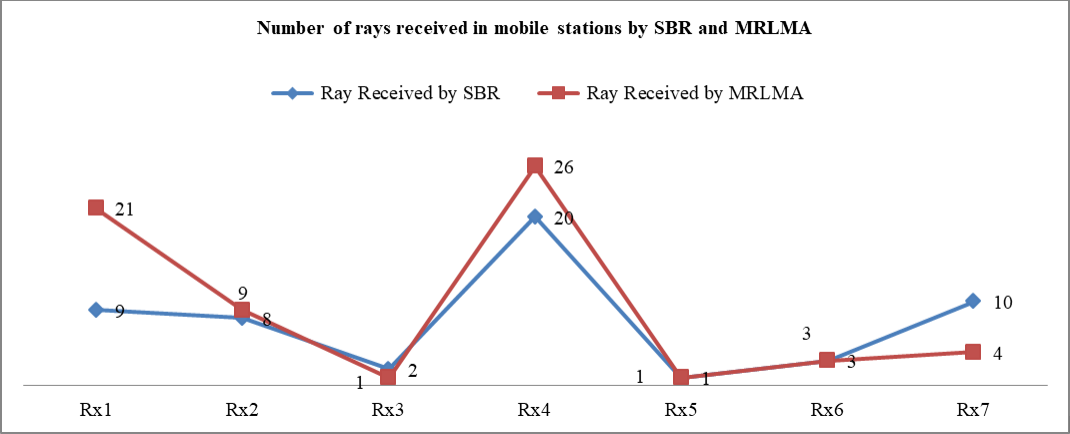
Comparison graph of the number of received rays in Rx by RL using SBR and MRLMA methods for scenario 2.

The Fig 10 shows that the coverage of using MRLMA method is better than SBR because the number of received rays by Rx is higher in the proposed method. From the scenario 2 both simulations data, it is shown that only 43.33% rays launching need to get better coverage in the proposed method compared to the conventional SBR, which also saves 39.94% simulation times.

**Fig 11:**
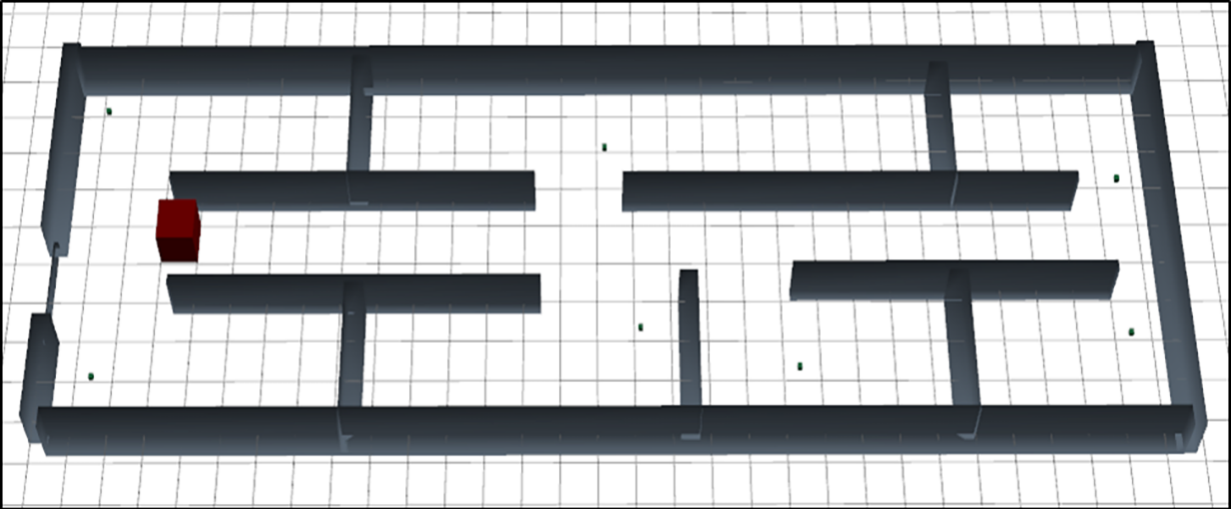
3D graphical representation of scenario 3.

The Fig 11 express the graphical design of the scenario 3 on the same layout as like Fig 3. One base station is placed on the left side of the floor. Seven Rx placed in the seven rooms.

**Fig 12:**
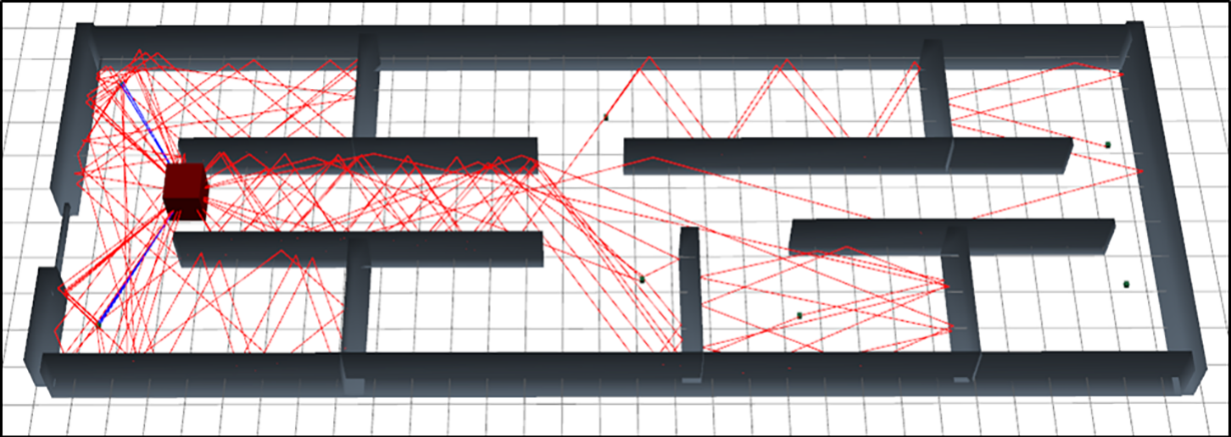
3D graphical view of scenario 3 simulation with SBR.

The Fig 12 represents the graphical output of the simulation of scenario 3 using SBR method. As per visual representation in this Fig 12 express that, it bearing larger numbers of reflections so it's path loss is very high.

Simulation output data for scenario 3 using SBR is presented in Table 6. As per the Table 6 data, the maximum and the minimum number of rays, that reached in the Rx are fifteen, and one. In total thirty-nine rays contributed among the six Rx where the total number of rays launching is 64800. Total simulation time is 7971.98 (ms).

**Table 6:** Simulation data for scenario 3 using SBR algorithm.

Simulation ID	Mobile Station ID	Ray Received	Simulation Time(MS)	Number of Ray Launched
MMM-FET-20180103150340	1d377c3d-8896-458f-8cc6e814cca51c72	5	7971.98	64800
	42daad4a-410e-4c29-86a378a6c35eaa37	1		
	9c42d834-7c27-46fc-a276-d12bba6fa3f4	8		
	9d589f5b-578d-4786-89b141c441f0ab03	8		
	bfb7d176-c9cb-433d-b0b2f450a96b7dba	15		
	e7a793c4-d0e3-48ed-a71fd3d121d2ad84	2		
**Total**		**39**	**7971.98**	**64800**

**Fig 13:**
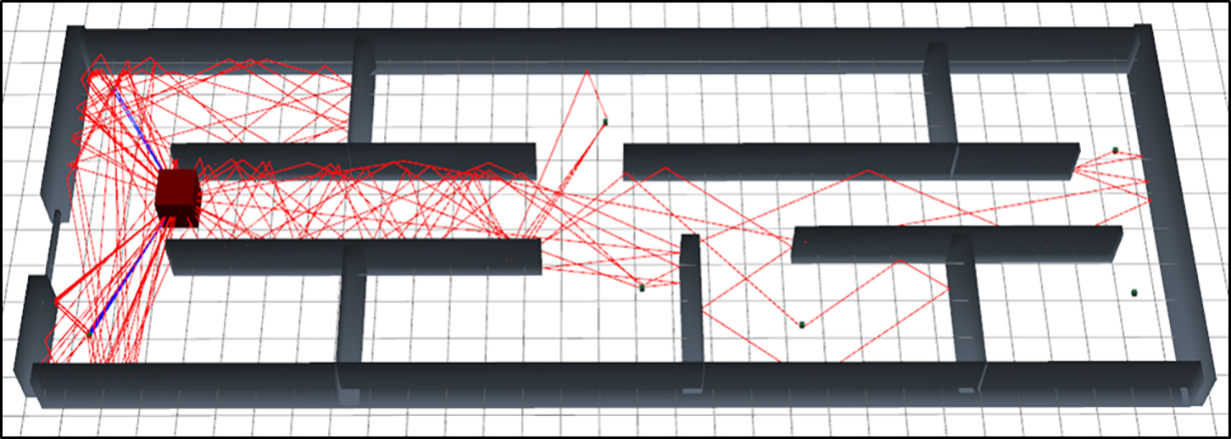
3D Graphical view of scenario 3 Simulation with MRLMA.

The Fig 13 represents the graphical output of the simulation of scenario 3 using MRLMA method. As per visual representation in this Fig 13 expresses that, it bearing fewer numbers of reflections so it's path loss is very low.

Simulation output data for the scenario 3 using MRLMA method is presented in Table 7. As per the Table 7 data, the maximum and the minimum number of rays, that reached in the Rx are twenty-seven, and two. In total sixty-four rays contributed among the six Rx where the total number of rays launching is 27720. Total simulation time is 5149.57 (ms).

**Table 7:** Simulation data for scenario 2 using MRLMA algorithm.

Simulation ID	Mobile Station ID	Ray Received	Simulation Time(MS)	Number of Ray Launched
MMU-FET-20180103150413	1d377c3d-8896-458f-8cc6-e814cca51c72	10	5149.57	27720
	42daad4a-410e-4c29-86a3-78a6c35eaa37	2		
	9c42d834-7c27-46fc-a276-d12bba6fa3f4	12		
	9d589f5b-578d-4786-89b1-41c441f0ab03	11		
	bfb7d176-c9cb-433d-b0b2-f450a96b7dba	27		
	e7a793c4-d0e3-48ed-a71f-d3d121d2ad84	2		
**Total**		**64**	**5149.57**	**27720**

**Fig 14:**
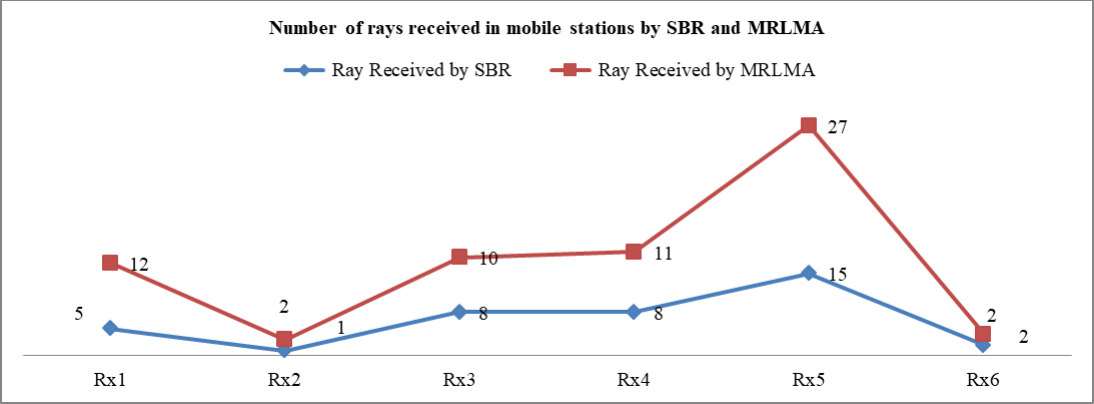
Comparison graph of the number of received rays in Rx by RL using SBR and MRLMA methods for scenario 3.

The Fig 14 shows that the coverage of using MRLMA method is far better than SBR because the number of received rays by Rx is higher in the proposed method. From the scenario 3 both simulations data, it is shown that only 35.06% rays launching need to get better coverage in the proposed method compared to the conventional SBR. It also saves 35.40% simulation times.

**Fig 15:**
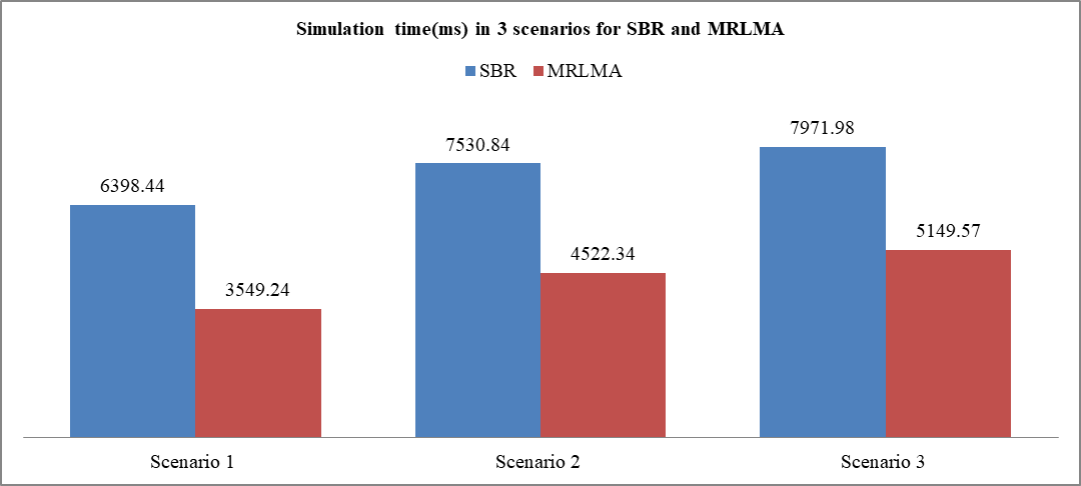
Comparison graph of simulation time that used in SBR and MRLMA methods for scenario 1–3.

The Fig 15 shows that the simulation time that used in MRLMA method is lower than SBR. Ray tracing is well known as a time-consuming technique due to its high simulation cost [26]. Base on the simulation time of 3 scenarios it proved that MRLMA saves 40% simulation times rather than SBR. This faster simulation is the remarkable contribution of MRLMA method.

**Fig 16:**
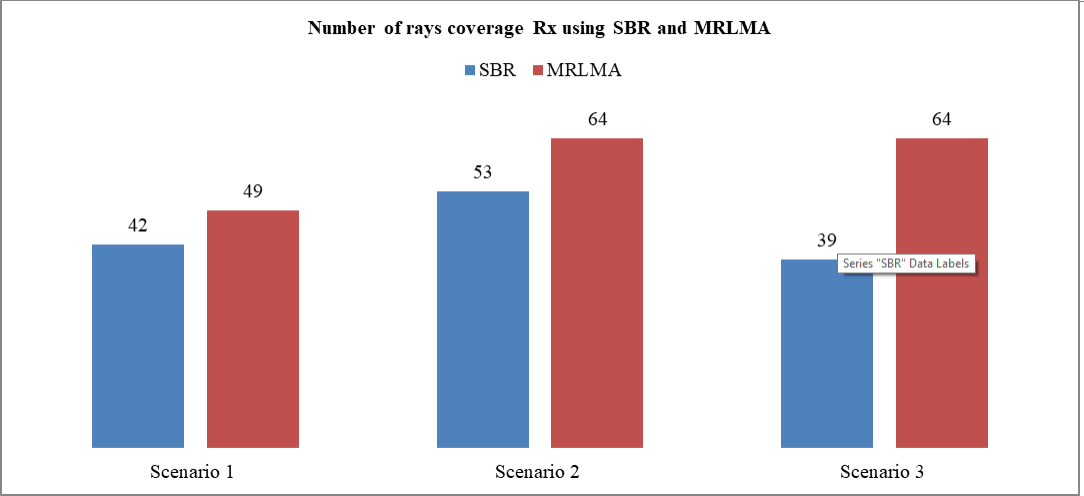
Comparison graph of number of rays coverage to Rx using SBR and MRLMA methods for scenario 1–3.

For the design of radio signal coverage, different mathematical radio propagation models are being used [27–29]. Coverage is the dynamic property of radio signal propagation because it depends on several factors. Fig 16 shows that the Rx rays coverage by using MRLMA method is higher than SBR. Based on the simulation coverage data of 3 scenarios it proved that MRLMA has 24.24% more good coverage rather than SBR method.

**Fig 17:**
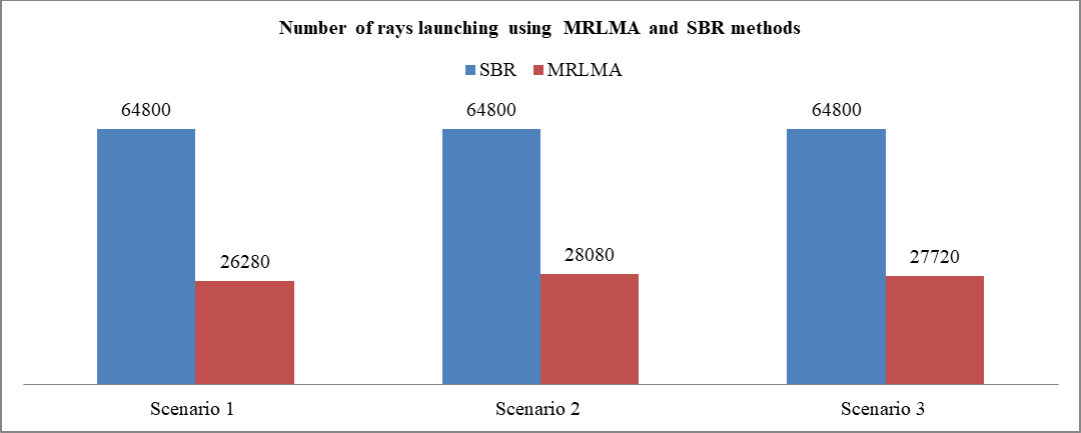
Comparison graph of the number of ray launching need for SBR and MRLMA methods for scenario 1–3.

Optimum ray launching plays vital role in ray tracing [30]. RL is the first phase of ray tracing. So proper utilization of MRLMA RL makes ray tracing more efficient and convenient. Fig 17 shows that, the number of ray launching need for MRLMA method is very much lower than SBR method. Based on the simulation’s data of 3 scenarios it proved that MRLMA saves 57.77% fewer rays to launch rather than SBR. To get good coverage using only rays 42.22% compare to SBR is the remarkable contribution of MRLMA method. So based on the good coverage, better simulation time, and best optimum ray launching MRLMA method demonstrated the best contribution in radio propagation prediction.

## Conclusion

In this paper, a new MRLMA method has been proposed for optimum ray launching, better simulation time and good coverage in the complex indoor environments. The MRLMA method is significantly advanced with respect to conventional ray launching technique like SBR because it launches the ray only in the Rx probable zoon with the high resolution where SBR launch ray in all directions. In specific three indoor complex scenarios have been selected and simulations have been done on these scenarios using the conventional and proposed algorithm to compare fully 3D ray launching data in the different dimension. It has been observed that proposed method simulation result is best with respect to conventional method result. The new method accomplishes an extraordinary gain in terms of computational proficiency, 40% decrease in simulation time and 57.77% reduction in the number of ray launch, leading to accurate results with good coverage.
